# Successful surgical intervention to ascending aortic aneurysm with dissection risk in a Patient with Cushing's syndrome and pituitary operation

**DOI:** 10.1186/1749-8090-10-S1-A229

**Published:** 2015-12-16

**Authors:** Ali Gürbüz, Ufuk Yetkin, Orhan Gökalp, Şahin İşcan, Köksal Dönmez, Nagihan Karahan

**Affiliations:** 1 Department of Cardiovascular Surgery, Katip Celebi University Izmir Ataturk Training and Research Hospital, Izmir, Turkey

## Background/Introduction

Patients with serious medical records due to chronic diseases have the chance of early identification of additional diseases and fast and safe surgical intervention if needed.

## Aims/Objectives

Our case was 44 year-old female patient. She had exertional chest pain for two months. She had pituitary surgery for Cushing's syndrome a year ago, in his medical history.

## Method

For investigation of her complaints transthoracic echocardiography, thoracoabdominal computerized tomography with contrast and coronary angiography combined with thoracic aortography revealed an aortic aneurysm with 55 mm in diameter, beginning 4 cm distally from aortic root and ending prior to arc of aorta. Surgical intervention was planned in Cardiology and Cardiovascular surgery council due to patient's risk of dissection and/or rupture.

## Results

Preoperative endocrinology consultation resulted with midnight cortisol level of 2,98 and cortisol level in 24 hours urine sample as 119 mcg/day. Pituitary MR screening was normal. With diagnosis of Subclinical Cushing (pseudoCushing) and control plan 6 months later, operation was approved. Under general anesthesia, median sternotomy was performed. Arterial cannulation was performed from small curvature of arcus aorta. Supracoronary Ascending aorta was replaced by 24 mm Ultramax double velour graft by using cardiopulmonary bypass. Patient recovered uneventfully. She is still followed up by endocrinology and our departments'' outpatient clinics.

## Discussion/Conclusion

Patients with Cushing's syndrome and ascending aorta aneurysm must be investigated by a multidisciplinary approach, due to risk of malign hypertension and connective tissue defects, which may cause dissection and rupture. Patients with operation indication may undergo surgery with low mortality rates by taking required measures, as in our case.

## Consent

Written informed consent was obtained from the patient for publication of this Case report and any accompanying images. A copy of the written consent is available for review by the Editor-in-Chief of this journal.

